# Prevalence of Autism Spectrum Disorder Severity Levels From the Fifth Edition of the Diagnostic and Statistical Manual (DSM-5) in the Autism and Developmental Disabilities Monitoring Network

**DOI:** 10.1007/s10803-026-07292-6

**Published:** 2026-03-24

**Authors:** Lauren A. Russell, Sarah C. Tinker, Kelly A. Shaw, Matthew J. Maenner, Monica Dirienzo, Anne V. Kirby, Ellen M. Howerton, Sandra B. Vanegas, Maya Lopez

**Affiliations:** 1 National Center on Birth Defects and Developmental Disabilities, Centers for Disease Control and Prevention, 4770 Buford Hwy NE, S106-4, Atlanta, GA 30341, USA; 2 Department of Occupational and Recreational Therapies, University of Utah, Salt Lake City, UT, USA; 3 Department of Epidemiology, Johns Hopkins Bloomberg School of Public Health, Baltimore, MD, USA; 4 Steve Hicks School of Social Work, University of Texas at Austin, Austin, TX, USA; 5 University of Arkansas for Medical Sciences, Little Rock, AR, USA

**Keywords:** Autism spectrum disorder, Severity level, Levels of support, DSM-5

## Abstract

**Purpose:**

The fifth edition of the Diagnostic and Statistical Manual of Mental Disorders (DSM-5) introduced severity level specifiers for autism spectrum disorder (ASD) with minimal description of the criteria for categorizing three levels of severity (1 to 3, with 3 being the most “severe”). The objective of the analysis was to assess the prevalence of ASD severity levels using population-based surveillance data.

**Methods:**

We analyzed severity level data on children with ASD ages 4- and 8-years-old in 2018 and 2020 in the multisite Autism and Developmental Disabilities Monitoring (ADDM) Network. Prevalence of any documented severity level and of each individual level were calculated overall and by demographic characteristics. Prevalence ratios adjusted for sex, race/ethnicity, age, intellectual disability, ADDM surveillance year, and site (aPRs) and 95% confidence intervals (CIs) were used for comparisons.

**Results:**

Less than half (40.4%) of children with documented ASD diagnoses had any severity level specified in their records, with wide variation by site (4.8%-73.2%). Severity levels were more common in records of children aged 4, in surveillance year 2020, and more often missing in non-Hispanic Black children and from records also missing information on intellectual disability (ID). Higher prevalence of more severe (level 3) ASD was observed among non-Hispanic Black children, children aged 4 years, children in 2020, and children with ID.

**Conclusion:**

Utilization of the DSM-5’s severity levels by community professionals varied widely, limiting their potential utility in identifying needed services and supports for children with ASD.

The fifth edition of the Diagnostic and Statistical Manual of Mental Disorders (DSM-5), published in 2013, defined autism spectrum disorder (ASD) as a disorder characterized by difficulty in social communication and restricted interests or repetitive behaviors (APA, 2013). A new feature for ASD in this edition was severity levels, defined by levels of needed support in these two diagnostic domains and are described as “requiring support” (Level 1), “requiring substantial support” (Level 2), and “requiring very substantial support” (Level 3) (Supplementary Table 1). These specifiers were intended to help clinicians describe the level of support an individual with ASD requires in daily functioning.

The DSM-5 introduced severity specifiers for ASD—and for most disorders—as part of a broader initiative by the American Psychiatric Association to encourage dimensional assessments, despite opposition from some members of the DSM-5 Neurodevelopmental Disorders Workgroup and autistic self-advocates. (Kapp & Ne’eman, 2020; Montanari, 2023). In addition, the severity levels were not validated ahead of publication (Lord, 2015). As described in several reviews, it was not specified if clinicians should assign severity levels based only on ASD-specific symptoms or by also considering co-occurring symptoms and impairments (Evers et al., 2021; Lord & Bishop, 2015; Mazurek et al., 2019; Mehling & Tassé, 2016; Weitlauf et al., 2014). There was no change regarding severity levels in the latest DSM update, the DSM-5 Text Revision (DSM-5-TR), released in 2022 (APA, 2022). It is not known how often community practitioners are assigning severity levels as part of ASD diagnosis or whether they are applied consistently.

Analyses exploring the reliability of severity levels have shown mixed findings. A study found poor inter-provider agreement in the assignment of severity levels, with many clinicians indicating that “they were ‘unsure’ of the level of support required by the child” (Taylor et al., 2017). This finding aligns with work showing that the application of ASD severity varies by clinician (Lord et al., 2012; Weitlauf et al., 2014). A range of associations were found between previously established measures of ASD functioning and the new DSM severity levels (Ellison et al., 2019; Hong & Matson, 2021; Mazurek et al., 2019; Weitlauf et al., 2014). Some studies (Gardner et al., 2018, 2023; Mazurek et al., 2019) reported a strong relationship between severity levels and measures of cognitive functioning, while Craig et al. (2017) highlighted the complexity of this relationship, noting that cognitive functioning can influence severity but is not the sole determinant.

Understanding usage patterns is important, as inconsistent or infrequent documentation may limit the utility of severity levels in informing service planning, eligibility decisions, and clinical communication. To address this gap, we analyzed population-based surveillance data from the Autism and Developmental Disabilities Monitoring (ADDM) Network to assess how frequently DSM-5 severity levels were documented and whether patterns varied by demographic and clinical characteristics.

## Methods

### Data Source

This analysis uses cross-sectional data from the ADDM Network surveillance years 2018 and 2020, the two most recent years of data that contained severity levels at the time of article submission. The ADDM Network conducts population-based surveillance of 4 and 8-year-old children with ASD at selected sites across the United States. Records-based surveillance methodology is used by the ADDM Network in which staff review records from medical, education, and other service providers, as previously described (Maenner et al., 2021; Maenner et al., 2023; Shaw et. al, 2021; Shaw et al., 2023).

### Population

In these years, a child met the ADDM case definition of ASD if they were aged 4 or 8 years, lived at least one day in the surveillance area during the study year (parts of Arizona, Arkansas, California, Georgia, Maryland, Minnesota, Missouri, New Jersey, Tennessee, Utah, and Wisconsin) and had a diagnostic statement, special education eligibility, or International Classification of Diseases code indicating ASD documented in their records. These children were included in our study if they had at least one comprehensive evaluation file in their records, where severity levels are typically documented.

### Measures

Information from comprehensive evaluations of children with ASD were collected, including severity levels. Severity levels (1, 2, or 3) and the associated domain social communication [SC], restrictive/repetitive behavior [RRB], or no domain specified [NDS] were abstracted from each evaluation when available. Other data collected from records included sociodemographic information (e.g., sex, race/ethnicity) and co-occurring intellectual disability (ID) status, defined as a child having an IQ score < = 70 or qualified professional’s statement of ID on their most recent cognitive test.

### Data Analysis

**Fig. 1:**
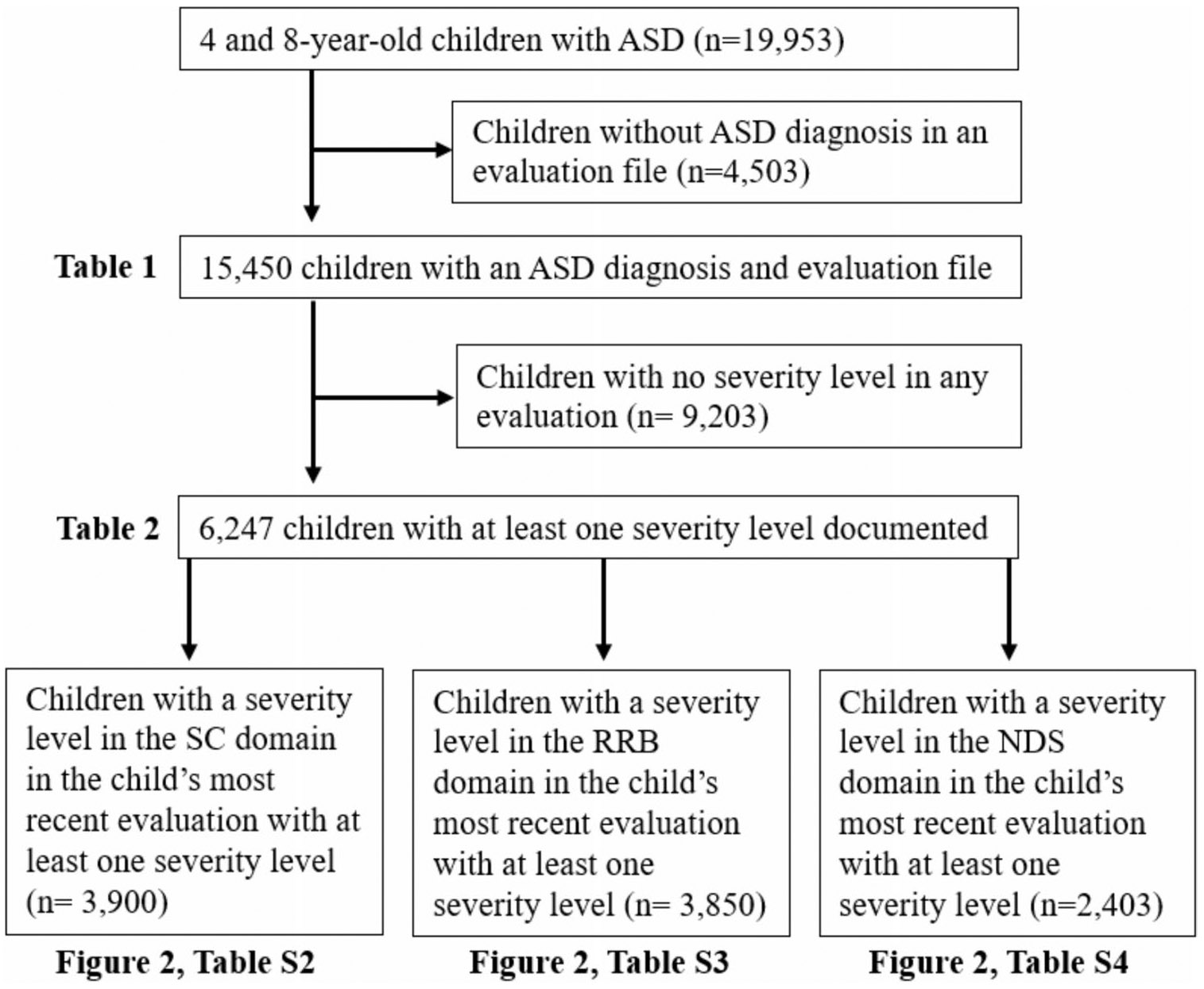
Flow chart of inclusion and exclusion criteria for each step of analysis

Children aged 4 and 8 years from 2018 and 2020 with a documented ASD diagnosis in an evaluation from a health or education source were included in the analysis (Fig. 1). If a child had at least one severity level in any domain (SC, RRB, no domain specified [NDS]) on any evaluation record, they were considered to have a severity level. The remaining children were considered to not have a severity level. The prevalence of these two categories were analyzed by site, sex, race/ethnicity (American Indian/Alaska Native, Asian, Black, Multiracial, White, Hispanic, unknown), and co-occurring ID (Yes, No, and Unknown). Persons of Hispanic origin might be of any race but are categorized as Hispanic; all racial groups are non-Hispanic. Children with no IQ tests in their records were categorized as Unknown ID status. Adjusted prevalence ratios (aPRs) with 95% confidence intervals (CIs) were calculated using a Poisson regression model with robust standard errors including all covariates described above.

Of the children with at least one severity level, we looked at the distribution of levels across the SC and RRB domains and NDS. This analysis was restricted to the subset of children with documented severity, and no imputation or weighting was applied. The prevalence of each severity level in all three domains was calculated for each sociodemographic characteristic. Because it was possible to record multiple levels in the same domain in a single evaluation and/or on the same date, if there were discordant levels, the lowest level (i.e., least severe) was used. Only 1% of the children with a severity level required this selection process. Adjusted prevalence ratios (aPRs) with 95% confidence intervals (CIs) were calculated using a Poisson regression model including all other covariates in the tables to assess statistical significance (Tables S2, S3, S4).

Analyses were performed in R Studio version 4.1.2.

## Results

### Overall Prevalence

Of the 15,450 children that had with autism diagnosis and a comprehensive evaluation in their records, most were male (79.1%) and white (52.0%) (Table 1). The overall prevalence of having any severity level was 40.4% (Table 1) and ranged widely by site (4.8% in New Jersey to 73.2% in California). There were no significant differences in the adjusted prevalence of having a severity level by sex. Non-Hispanic Black children (31.9%) were less likely to have a severity level than non-Hispanic White children (43.5%) (aPR = 0.91 [95% CI: 0.85, 0.98]). Severity levels were less prevalent in eight-year-olds (33.7%) than four-year-olds (48.6%, aPR = 0.69 [95% CI: 0.66, 0.73]). Prevalence of severity levels did not differ significantly between children with ID (41.6%) and without ID (49.1%, aPR = 0.97 [95% CI: 0.92, 1.04]); however, severity levels were less prevalent in children without documentation of ID status (30.3%; aPR using children without ID as the reference group = 0.66 [95% CI:0.62, 0.70]). Severity levels were more prevalent in surveillance year 2020 (44.7%) than in surveillance year 2018 (35.1%, aPR = 1.23 [95% CI: 1.16, 1.30]).

### Severity Level Distributions Across Domains

Of the 6,247 children with at least one severity level documented, about a third only had a level with no domain specified, labeled NDS (33.2%, Table 2). Most children had at least one level in the SC or RRB domains (66.5% and 65.7%, respectively).

### Subgroup Comparisons of Those With at Least One Severity Level

**Fig. 2:**
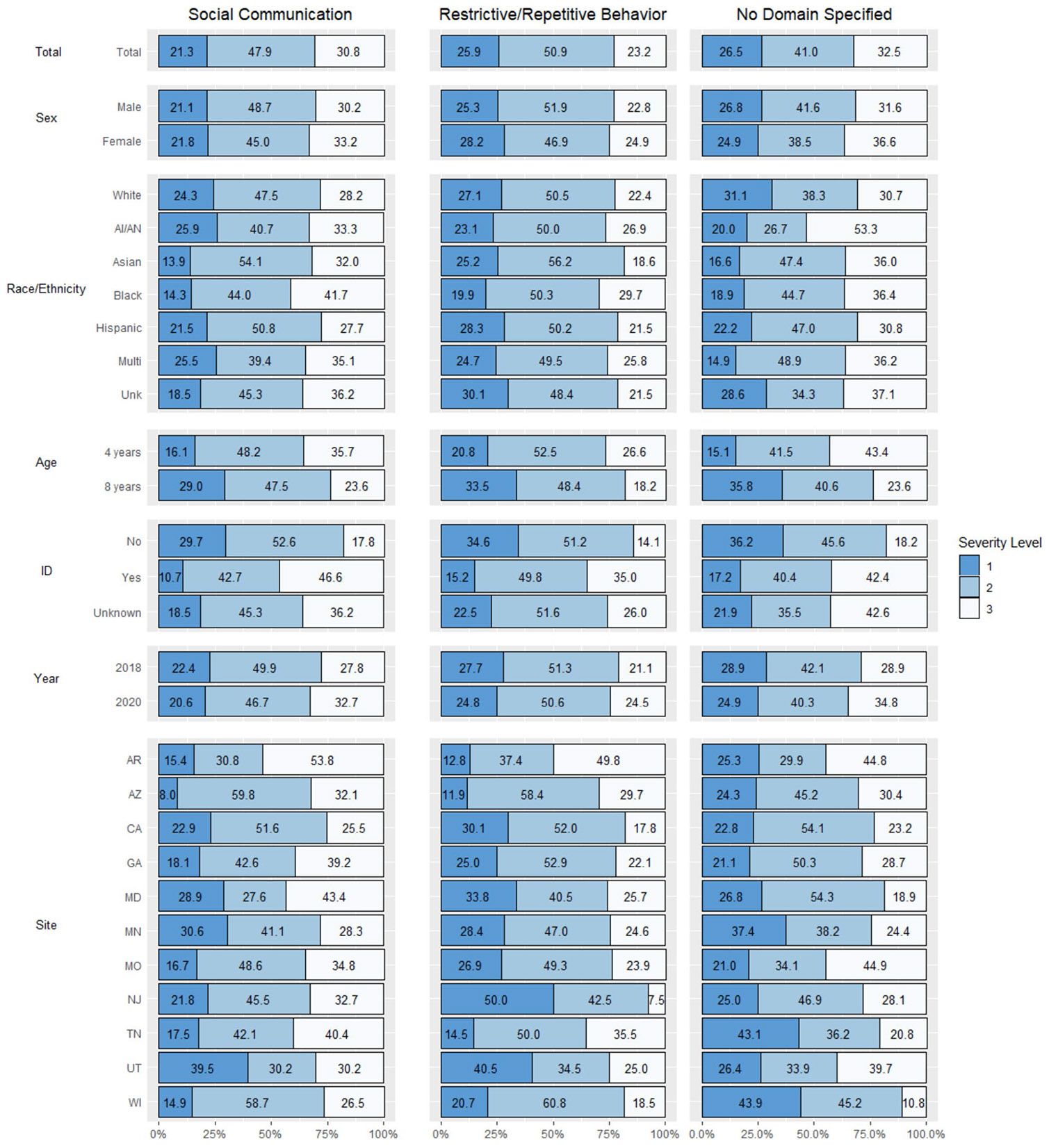
Prevalence of each autism spectrum disorder severity level across Social Communication and Restrictive/Repetitive Behavior domains and No Domain Specified by selected demographic characteristics, Autism and Developmental Disabilities Monitoring (ADDM) Network, 2018 and 2020. All Race/ethnicity categories are non-Hispanic except for Hispanic category; AI/AN = American Indian/Alaskan Native, Multi = Multiracial, Unk = Unknown

Among children with at least one severity level documented, level 2 was the most common rating across domains (41.0–50.9%), with fewer level 1 (21.3–26.5%) and level 3 (23.2–32.5%) assignments (Fig. 2, Tables S2, S3, S4). In general, the patterns of severity level distribution by demographic factors were similar for all domains. Non-Hispanic Asian children were less likely than non-Hispanic White children to have a level 1 SC or NDS severity (aPR = 0.55 [95% CI: 0.41, 0.75] and aPR = 0.63 [95% CI: 0.43, 0.92], respectively). Compared to non-Hispanic White children, non-Hispanic Black children were less likely to have a level 1 (aPR = 0.69 [95% CI: 0.55, 0.88]) and more likely to have a level 3 (aPR = 1.22 [95% CI: 1.04, 1.43) SC severity and less likely to have a level 1 in NDS (aPR = 0.70 [95% CI: 0.55, 0.89]).

In all domains, children 8 years of age were more likely to be assigned a severity level 1 (SC aPR = 1.62 [95% CI: 1.40, 1.87]; RRB aPR = 1.51 [95% CI: 1.33, 1.73]; NDS aPR = 1.97 [95% CI: 1.64, 2.38]) and less likely to be assigned a severity level 3 (SC aPR = 0.69 [95% CI: 0.61, 0.79]; RRB aPR = 0.71 [95% CI: 0.61, 0.82]; NDS aPR = 0.67 [95% CI: 0.57, 0.78]) compared to children aged 4 years.

Children with ID were more than twice as likely to be assigned Level 3 severity across all domains compared to those without ID. Nearly half of children with ID had SC severity level 3 (46.6%), compared to only 17.8% of children without ID (aPR = 2.44 [95% CI: 2.11, 2.81]), while the prevalence of SC severity level 1 was almost three times lower among children with ID (10.7%) compared to children without ID (29.7%) (aPR = 0.40 [95% CI: 0.33, 0.49]). This pattern was also observed for the RRB domain; more than twice as many children with ID had a level 3 severity (35.0%) as children without ID (14.1%) (aPR = 2.23 [95% CI: 1.90, 2.63]), and half as many children with ID had a level 1 severity (15.2%) compared to children without ID (34.6%) (aPR = 0.50 [95% CI: 0.42, 0.59]). Similarly, for NDS approximately twice as many children with ID had a level 3 severity (42.4%) as children without ID (18.2%) (aPR = 2.14 [95% CI: 1.76, 2.60]), and about half as many children with ID had a level 1 severity (17.2%) compared to children without ID (36.2%) (aPR = 0.55 [95% CI: 0.44, 0.69]). Children with missing information on ID showed similar, though attenuated, patterns of severity level assignment as seen for children with ID in both domains and NDS.

Among those with at least one severity level in their records, children included in surveillance year 2020 were more likely to be assigned a severity level of 3 in the SC and RRB domains and NDS (SC aPR = 1.23 [95% CI: 1.08, 1.40]; RRB aPR = 1.24 [95% CI: 1.06, 1.44]; NDS aPR = 1.28 [95% CI: 1.10, 1.50]) and less likely to be assigned a severity level of 1 in the RRB domain and NDS (aPR = 0.86 [95% CI: 0.75, 0.99] and aPR = 0.81 [95% CI: 0.69, 0.96], respectively).

Distribution of severity levels varied widely by site, particularly for the RRB domain. The Arkansas site had the highest prevalence of level 3 severity, accounting for 53.8%, 49.8%, and 44.8% of levels assigned in the SC and RRB domains and NDS, respectively. The New Jersey site had the lowest prevalence of RRB level 3 severity (7.5%), with half (50.0%) assigned to RRB severity level 1.

## Discussion

Our analyses of population-based data on the prevalence of severity levels 5 to 7 years after their introduction in the DSM-5 show that less than half of children had any documentation of a severity level in their records and that there was considerable variability across communities, age groups, and surveillance years. The distribution of specific severity levels within the SC and RRB domains also showed considerable variability, particularly by age, ID status, surveillance year, and site. Lack of uniform implementation of severity levels across multiple communities in the United States limits the utility of the descriptor.

The relatively higher prevalence of severity levels in 4 versus 8-year-olds and in 2020 versus 2018 suggest increasing use of severity levels over time; however, more than half of children in either surveillance year or either age group lacked an assigned severity level. After the DSM-5 introduced severity levels for ASD in 2013, there was considerable debate about their validity and inter-rater reliability (Ellison et al. 2019; Evers et al. 2021; Gardner et al. 2018; Gardner et al. 2023; Kapp & Ne’eman, 2020; Lord & Bishop, 2015; Mazurek et. al, 2019; Mehling & Tasse, 2016; Taylor et. al, 2017; Weitlauf et al., 2014). We could not assess whether these concerns resulted in reduced clinician use of severity specifiers. Our findings could, however, reveal a need for education and training or a need for further refinement of these categories into something that provides clinical utility to help identify supports and services, or to guide prognosis or expectations for the future.

The results reveal clear age-related differences in severity levels, with older children more often rated as Level 1 and less frequently as Level 3. This trend may reflect that children identified later tend to be less severely affected, often less likely to have co-occurring intellectual disabilities (Maenner et al., 2021, 2023). This later identification may contribute to the observed differences in severity ratings by age group.

Children with ASD and ID were more likely to be classified as having higher support needs than children with ASD without ID, suggesting that ID may be a contributing factor to clinicians when assigning ASD severity labels. Greater cognitive impairment often corresponds with greater functional support needs, which may explain the higher severity classification observed in this group (Lord et al., 2022). However, even among children with ID, fewer than half were assigned to the highest severity level in either domain. Records missing ID documentation were more likely to lack severity specifiers, indicating that more complete comprehensive evaluations may be more likely to include both types of information. Our observations are consistent with other studies that have reported higher severity levels associated with lower IQ scores (Gardner et al., 2018, 2023; Mazurek et al., 2019). A significant challenge to implementation and utilization of severity levels is lack of guidance in the DSM-5 as to whether a provider should assign severity exclusively from symptoms related to ASD or should factor in symptoms from co-occurring conditions like ID (Evers et al., 2021; Lord & Bishop, 2015; Mazurek et al., 2019; Mehling & Tassé, 2016; Weitlauf et al., 2014).

We also observed that non-Hispanic Black children had higher levels of severity of ASD compared to non-Hispanic White children, even after adjusting for ID. ADDM data have consistently shown a higher prevalence of ID among non-Hispanic Black children with ASD compared to non-Hispanic White children with ASD (Baio et al., 2018; Maenner et al., 2020, 2021, 2023; Shaw et al., 2022). The higher severity levels of ASD observed in non-Hispanic Black children compared to non-Hispanic White children may be partially attributable to the increased prevalence of co-occurring intellectual disability among non-Hispanic Black children, which could influence severity assignment even after adjustment. Additionally, systemic barriers may lead to under-identification of non-Hispanic Black children without co-occurring ID, as disparities in access to services and socioeconomic factors can hinder timely diagnosis and evaluation (Maenner et al., 2020; Shaw et al., 2022).

Wide variation in use and assignment of severity levels across sites suggest a lack of standard of practice for determining a child’s level of severity. This may be due to differences in diagnostic training, clinical protocols, or documentation practices. The DSM-5 specifies that “the descriptive severity categories should not be used to determine eligibility for provision of services. Indeed, individuals with relatively better skills overall may experience different or even greater psychosocial challenges. Thus, service needs can only be developed at an individual level and through discussion of personal priorities and targets” (APA, 2013). Providers may therefore question the utility of severity levels defined by support needs, when the DSM also advises that service needs be determined on an individual basis. There is at least one documented instance of severity levels being used to determine eligibility for services: Australia requires at least a level 2 designation to receive their National Disability Insurance Scheme (NDIS) (National Disability Insurance Agency, 2022). It is unknown whether and how often severity levels are used by U.S. service systems.

The degree to which autistic people find utility in the severity levels is unclear. Some who had been diagnosed under the DSM-IV with Asperger’s disorder found the Asperger’s descriptor helpful in understanding themselves and in describing their needs, and they reported concern when it was removed from the DSM-5 (Kapp & Ne’eman, 2020). The severity levels introduced in the DSM-5 could provide a similar type of descriptor that some autistic people find useful. However, other autistic people have expressed concern about the use of severity levels because they might be used to limit care access or to inappropriately group people with very different types of support needs (Kapp & Ne’eman, 2020). Similar concerns have been expressed in other attempts at grouping functioning in ASD, notably with the introduction of the term “profound” ASD, coined by the Lancet Commission on the Future and Care and Clinical Research in Autism (Kapp, 2023; Kripke-Ludwig, 2023; Lord et al., 2022; Pukki et al., 2022).

## Limitations

The findings of this study are subject to several limitations. First, our analyses relied on population-based data collected through the ADDM Network, which depends on the completeness and quality of available records. As highlighted in our methodology, variability in the ascertainment and reporting of ASD severity levels and comorbid conditions such as ID may have influenced the representativeness of our sample. Specifically, missing data due to incomplete evaluations for ID or ASD severity could introduce bias, potentially affecting the generalizability of our findings to the broader population of children with ASD. Additionally, our findings are specific to 4- and 8-year-old children and may not generalize to other age groups.

Some children had multiple severity levels in the same domain assigned during a single assessment, while others lacked assigned severity levels altogether. These inconsistencies underscore the complexity and variability in clinical practices and diagnostic reporting, which could impact the reliability of our conclusions. Furthermore, it is important to acknowledge that the geographically defined ADDM Network sites are not necessarily representative, which limits the extrapolation of our results to the entire populations of the states where these sites are located. These findings do not reflect children who did not have comprehensive evaluations in ADDM for surveillance years 2018 and 2020 or whose ASD diagnosis was missed. Finally, this is a cross-sectional study which does not allow the examination of longitudinal changes in individual severity levels. While our study provides valuable insights into the patterns and correlates of ASD severity levels among children, caution should be exercised in applying these findings broadly without considering the specific contexts and limitations of the data.

## Conclusion

In our analysis of population-based data from multiple sites, inclusion of ASD severity levels as described in the DSM-5 in clinical diagnostic records for ASD appears to be increasing. However, their prevalence overall is low and varied widely, as did assignment of specific severity levels, limiting their potential utility in identifying needed services and supports for children with ASD. This data may help guide community discussions on improving the use of severity levels and highlight the need for further research and clearer implementation strategies. Due to the range and heterogeneity of ASD symptoms and diversity of needs of autistic individuals, some sort of descriptor for severity may be useful for planning to provide services and supports to help individuals with ASD achieve their goals.

## Supplementary Material

SUP - Russell - Prevalence of Autism Spectrum Disorder Severity Levels From the Fifth Edition of the Diagnostic and Statistical Manual

**Supplementary Information** The online version contains supplementary material available at https://doi.org/10.1007/s10803-026-07292-6.

## Figures and Tables

**Table 1:** Prevalence of autism spectrum disorder severity level in children ages 4 and 8 years, by selected demographic characteristics, Autism and Developmental Disabilities Monitoring (ADDM) Network, 2018 and 2020

Total		At least one severity level		Adjusted prevalence ratio^1^ (95% Confidence interval)
	
	n (%)			
	
Total	15,450	6,247	(40.4)	
*Sex*				
Male	12,227	4,964	(40.6)	[Reference]
Female	3,219	1,281	(39.8)	0.98 (0.92, 1.04)
*Race/Ethnicity*				
Non-Hispanic White	8,027	3,494	(43.5)	[Reference]
Non-Hispanic AIAN^2^	99	42	(42.4)	0.97 (0.72, 1.32)
Non-Hispanic Asian	1,077	526	(48.8)	1.02 (0.93, 1.12)
Non-Hispanic Black	3,438	1,096	(31.9)	0.91 (0.85, 0.98)*
Hispanic	2011	818	(40.7)	0.93 (0.86, 1.01)
Non-Hispanic	343	140	(32.1)	0.95 (0.80, 1.12)
Multiracial				
Unknown	455	131	(28.8)	0.95 (0.80, 1.13)
*Age*				
4 years old	6,950	3,380	(48.6)	[Reference]
8 years old	8,500	2,867	(33.7)	0.69 (0.66, 0.73)*
*Intellectual Disability*				
No	5,666	2,780	(49.1)	[Reference]
Yes	4,420	1,840	(41.6)	0.97 (0.92, 1.04)
Unknown	5,364	1,627	(30.3)	0.66 (0.62, 0.70)*
*ADDM Surveillance year*				
2018	6,855	2,406	(35.1)	[Reference]
2020	8,595	3,841	(44.7)	1.23 (1.16, 1.30)*
*Site*				
Arkansas	1,023	402	(39.3)	[Reference]
Arizona	771	339	(44.0)	1.13 (0.98, 1.31)
California	2,244	1,643	(73.2)	1.75 (1.56, 1.96)*
Georgia	1,266	373	(29.5)	0.78 (0.68, 0.90)*
Maryland	1,126	341	(30.3)	0.81 (0.70, 0.94)*
Minnesota	764	369	(48.3)	1.23 (1.07, 1.42)*
Missouri	1,611	862	(53.5)	1.56 (1.38, 1.76)*
New Jersey	1,777	86	(4.8)	0.14 (0.11, 0.17)*
Tennessee	1,782	359	(20.1)	0.53 (0.46, 0.62)*
Utah	1,287	594	(46.2)	1.36 (1.19, 1.55)*
Wisconsin	1799	879	(48.9)	1.40 (1.24 1.58)*

* These 95% confidence intervals do not include 1

1 Adjusted for all other variables in the table

2 American Indian/Alaskan Native

**Table 2:** Prevalence of severity domain among children ages 4 and 8 years with autism spectrum disorder and at least one severity level, Autism and Developmental Disabilities Monitoring (ADDM) Network, 2018 and 2020

	Among children with at least one severity level (n = 6,247)	
Any instance of Domain Severity Level	n	%
Any Social Communication	4,157	(66.5)
Any Restrictive/Repetitive Behavior	4,106	(65.7)
Any level(s) categorized as NDS	2,538	(40.6)
*Only instance of one Domain Severity Level*		
Only has Social Communication	50	(0.8)
Only has Restrictive/Repetitive Behavior	10	(0.2)
Only has level(s) categorized as NDS	2,075	(33.2)
