# Supplementary material for: Prevalence of Autism Spectrum Disorder Severity Levels From the Fifth Edition of the Diagnostic and Statistical Manual (DSM-5) in the Autism and Developmental Disabilities Monitoring Network
Source: J Autism Dev Disord. Author manuscript; Available in PMC 2026 Jun 15. (PMC13267130; doi:10.1007/s10803-026-07292-6)
Supplement: SUP - Russell - Prevalence of Autism Spectrum Disorder Severity Levels From the Fifth Edition of the Diagnostic and Statistical Manual [file NIHMS2178608-supplement-SUP_-_Russell_-_Prevalence_of_Autism_Spectrum_Disorder_Severity_Levels_From_the_Fifth_Edition_of_the_Diagnostic_and_Statistical_Manual.docx]

Supplement

**Table S1.** Reproduction of “Severity levels for autism spectrum disorder (examples of level of support needs)” from the fifth edition of the Diagnostic and Statistical Manual or Mental Disorders-Text Revision (DSM-5-TR) (APA, 2022)

| Severity level | Social communication | Restricted, repetitive behaviors |
| --- | --- | --- |
| Level 1  “Requiring support” | Without supports in place, deficits in social communication cause noticeable impairments. Difficulty initiating social interactions, and clear examples of atypical or unsuccessful responses to social overtures of others. May appear to have decreased interest in social interactions. For example, a person who is able to speak in full sentences and engages in communication but whose to-and-fro conversation with others fails, and whose attempts to make friends are odd and typically unsuccessful. | Inflexibility of behavior causes significant interference with functioning in one or more contexts. Difficulty switching between activities. Problems of organization and planning hamper independence. |
| Level 2  “Requiring substantial support” | Marked deficits in verbal and nonverbal social communication skills; social impairments apparent even with supports in place; limited initiation of social interactions; and reduced or abnormal responses to social overtures from others. For example, a person who speaks simple sentences, whose interaction is limited to narrow special interests, and who has markedly odd nonverbal communication. | Inflexibility of behavior, difficulty coping with change, or other restricted/repetitive behaviors appear frequently enough to be obvious to the casual observer and interfere with functioning in a variety of contexts. Distress and/or difficulty changing focus or action. |
| Level 3  “Requiring very substantial support” | Severe deficits in verbal and nonverbal social communication skills cause severe impairments in functioning, very limited initiation of social interactions, and minimal response to social overtures from others. For example, a person with few words of intelligible speech who rarely initiates interaction and, when he or she does, makes unusual approaches to meet needs only and responds to only very direct social approaches. | Inflexibility of behavior, extreme difficulty coping with change, or other restricted/repetitive behaviors markedly interfere with functioning in all spheres. Great distress/difficulty changing focus or action. |

**Table S2.** Prevalence of each autism spectrum disorder severity level in the Social Communication domain by selected characteristics, Autism and Developmental Disabilities Monitoring (ADDM) Network, 2018 and 2020

|  | **Severity Level: Social Communication** | | | | **Adjusted Prevalence Ratio^1^**  **(95% Confidence Interval)** | |
| --- | --- | --- | --- | --- | --- | --- |
|  | Total | 1 | 2 | 3 | Severity Level 1 | Severity Level 3 |
|  | n | n (%) | | |  |  |
| **Total** | 3,900 | 830 (21.3) | 1,868 (47.9) | 1,202 (30.8) |  | |
| **Sex** |  | | | | | |
| Male | 3,079 | 650 (21.1) | 1,499 (48.7) | 930 (30.2) | Reference | |
| Female | 820 | 179 (21.8) | 369 (45.0) | 272 (33.2) | 1.10  (0.93, 1.30) | 1.08  (0.94, 1.23) |
| **Race/Ethnicity** |  | | | | | |
| Non-Hispanic White | 2,067 | 502 (24.3) | 982 (47.5) | 583 (28.2) | Reference | |
| Non-Hispanic American Indian/Alaska Native | 27 | 7  (25.9) | 11  (40.7) | 9  (33.3) | 1.14  (0.54, 2.42) | 1.22  (0.63, 2.36) |
| Non-Hispanic Asian | 353 | 49 (13.9) | 191 (54.1) | 113 (32.0) | 0.55  (0.41, 0.75)* | 1.22  (0.99, 1.50) |
| Non-Hispanic Black | 636 | 91 (14.3) | 280 (44.0) | 265 (41.7) | 0.69  (0.55, 0.88)* | 1.22  (1.04, 1.43)* |
| Hispanic | 624 | 134 (21.5) | 317 (50.8) | 173 (27.7) | 0.89  (0.72, 1.10) | 1.11  (0.93, 1.34) |
| Non-Hispanic Multiracial | 94 | 24 (25.5) | 37  (39.4) | 33 (35.1) | 0.96  (0.63, 1.47) | 1.37  (0.96, 1.97) |
| Unknown | 99 | 23 (23.2) | 50 (50.5) | 26 (26.3) | 0.96  (0.63, 1.46) | 0.88  (0.59, 1.30) |
| **Age** |  | | | | | |
| 4 years old | 2,322 | 373 (16.1) | 1,119 (48.2) | 830 (35.7) | Reference | |
| 8 years old | 1,578 | 457 (29.0) | 749 (47.5) | 372 (23.6) | 1.62  (1.40, 1.87)* | 0.69  (0.61, 0.79)* |
| **Intellectual Disability** |  | | | | | |
| No | 1,821 | 540 (29.7) | 957 (52.6) | 324 (17.8) | Reference | |
| Yes | 1,201 | 128 (10.7) | 513 (42.7) | 560 (46.6) | 0.40  (0.33, 0.49)* | 2.44  (2.11, 2.81)* |
| Unknown | 878 | 162 (18.5) | 398 (45.3) | 318 (36.2) | 0.63  (0.52, 0.76)* | 2.20  (1.86, 2.60)* |
| **ADDM Surveillance Year** |  | | | | | |
| 2018 | 1,506 | 337 (22.4) | 751 (49.9) | 418 (27.8) | Reference | |
| 2020 | 2,394 | 493 (20.6) | 1,117 (46.7) | 784 (32.7) | 0.88  (0.76, 1.02) | 1.23  (1.08, 1.40)* |
| **Site** |  | | | | | |
| Arkansas | 247 | 38 (15.4) | 76  (30.8) | 133  (53.8) | Reference | |
| Arizona | 224 | 18  (8.0) | 134 (59.8) | 72 (32.1) | 0.46  (0.26, 0.81)* | 0.68  (0.51, 0.91)* |
| California | 1,406 | 322 (22.9) | 725 (51.6) | 359 (25.5) | 1.36  (0.96, 1.91) | 0.59  (0.47, 0.72)* |
| Georgia | 204 | 37 (18.1) | 87  (42.6) | 80 (39.2) | 1.49  (0.94, 2.36) | 0.63  (0.47, 0.83)* |
| Maryland | 76 | 22 (28.9) | 21  (27.6) | 33 (43.4) | 2.22  (1.30, 3.78)* | 0.75  (0.51, 1.11) |
| Minnesota | 265 | 81 (30.6) | 109 (41.1) | 75 (28.3) | 1.76  (1.19, 2.59)* | 0.62  (0.46, 0.83)* |
| Missouri | 138 | 23 (16.7) | 67  (48.6) | 48 (34.8) | 1.09  (0.65, 1.84) | 0.63  (0.45, 0.88)* |
| New Jersey | 55 | 12 (21.8) | 25  (45.5) | 18 (32.7) | 1.15  (0.60, 2.20) | 0.80  (0.48, 1.31) |
| Tennessee | 228 | 40 (17.5) | 96  (42.1) | 92 (40.4) | 1.47  (0.94, 2.31) | 0.65  (0.50, 0.86)* |
| Utah | 324 | 128 (39.5) | 98  (30.2) | 98 (30.2) | 2.07  (1.43, 3.00) | 0.66  (0.50, 0.88)* |
| Wisconsin | 733 | 109 (14.9) | 430 (58.7) | 194 (26.5) | 1.06  (0.73, 1.55) | 0.45  (0.36, 0.57)* |

^*^ These 95% confidence intervals do not include 1

^1^ Adjusted for all other variables in the table

**Table S3.** Prevalence of each autism spectrum disorder severity level in the Restricted/Repetitive Behavior domain by selected characteristics, Autism and Developmental Disabilities Monitoring (ADDM) Network, 2018 and 2020

|  | **Severity Level: Restrictive/Repetitive Behavior** | | | | | **Adjusted Prevalence Ratio^1^ (95% Confidence Interval)** | |
| --- | --- | --- | --- | --- | --- | --- | --- |
|  | Total | 1 | 2 | 3 | Severity Level 1 | | Severity Level 3 |
|  | n | n (%) | | |  | |  |
| **Total** | 3,850 | 998 (25.9) | 1,958 (50.9) | 894 (23.2) |  | | |
| **Sex** |  |  |  |  |  | |  |
| Male | 3,038 | 768 (25.3) | 1,578 (51.9) | 692 (22.8) | Reference | | |
| Female | 811 | 229 (28.2) | 380 (46.9) | 202 (24.9) | 1.17  (1.01, 1.36)* | | 1.08  (0.92, 1.27) |
| **Race/Ethnicity** |  | | | | | | |
| Non-Hispanic White | 2,039 | 552 (27.1) | 1,030 (50.5) | 457 (22.4) | Reference | | |
| Non-Hispanic American Indian/Alaska Native | 26 | 6  (23.1) | 13  (50.0) | 7  (26.9) | 0.90  (0.40, 2.03) | | 1.22  (0.57, 2.58) |
| Non-Hispanic Asian | 349 | 88  (25.2) | 196 (56.2) | 65  (18.6) | 0.85  (0.67, 1.07) | | 0.97  (0.74, 1.27) |
| Non-Hispanic Black | 632 | 126 (19.9) | 318 (50.3) | 188 (29.7) | 0.84  (0.68, 1.03) | | 1.17  (0.97, 1.41) |
| Hispanic | 618 | 175 (28.3) | 310 (50.2) | 133 (21.5) | 0.97  (0.81, 1.17) | | 1.16  (0.94, 1.43) |
| Non-Hispanic Multiracial | 93 | 23  (24.7) | 46  (49.5) | 24  (25.8) | 0.84  (0.55, 1.28) | | 1.29  (0.85, 1.97) |
| Unknown | 93 | 28  (30.1) | 45  (48.4) | 20  (21.5) | 1.12  (0.76, 1.65) | | 0.94  (0.60, 1.48) |
| **Age** |  | | | | | | |
| 4 years old | 2,303 | 480 (20.8) | 1,210 (52.5) | 613 (26.6) | Reference | | |
| 8 years old | 1,547 | 518 (33.5) | 748 (48.4) | 281 (18.2) | 1.51  (1.33, 1.73)* | | 0.71  (0.61, 0.82)* |
| **Intellectual Disability** |  | | | | | | |
| No | 1,798 | 623 (34.6) | 921 (51.2) | 254 (14.1) | Reference | | |
| Yes | 1,185 | 180 (15.2) | 590 (49.8) | 415 (35.0) | 0.50  (0.42, 0.59)* | | 2.23  (1.90, 2.63)* |
| Unknown | 867 | 195 (22.5) | 447 (51.6) | 225 (26.0) | 0.66  (0.55, 0.79)* | | 1.94  (1.59, 2.35)* |
| **ADDM Surveillance Year** |  | | | | | | |
| 2018 | 1,475 | 408 (27.7) | 756 (51.3) | 311 (21.1) | Reference | | |
| 2020 | 2,375 | 590 (24.8) | 1,202 (50.6) | 583 (24.5) | 0.86  (0.75, 0.99)* | | 1.24  (1.06, 1.44)* |
| **Site** |  | | | | | | |
| Arkansas | 243 | 31  (12.8) | 91  (37.4) | 121 (49.8) | Reference | | |
| Arizona | 219 | 26  (11.9) | 128 (58.4) | 65  (29.7) | 0.87  (0.52, 1.47) | | 0.66  (0.48, 0.90)* |
| California | 1,403 | 423 (30.1) | 730 (52.0) | 250 (17.8) | 2.14  (1.48, 3.11)* | | 0.44  (0.35, 0.56)* |
| Georgia | 204 | 51  (25.0) | 108 (52.9) | 45  (22.1) | 2.23  (1.42, 3.50)* | | 0.39  (0.28, 0.56)* |
| Maryland | 74 | 25  (33.8) | 30  (40.5) | 19  (25.7) | 2.94  (1.73, 5.00)* | | 0.49  (0.30, 0.80)* |
| Minnesota | 264 | 75  (28.4) | 124 (47.0) | 65  (24.6) | 1.98  (1.30, 3.02)* | | 0.59  (0.43, 0.80)* |
| Missouri | 134 | 36  (26.9) | 66  (49.3) | 32  (23.9) | 2.08  (1.28, 3.39)* | | 0.49  (0.33, 0.73)* |
| New Jersey | 40 | 20  (50.0) | 17  (42.5) | 3  (7.5) | 3.09  (1.75, 5.44)* | | 0.20  (0.06, 0.64)* |
| Tennessee | 228 | 33  (14.5) | 114 (50.0) | 81  (35.5) | 1.39  (0.85, 2.28) | | 0.63  (0.47, 0.84)* |
| Utah | 316 | 128 (40.5) | 109 (34.5) | 79  (25.0) | 2.70  (1.81, 4.03)* | | 0.59  (0.44, 0.80)* |
| Wisconsin | 725 | 150 (20.7) | 441 (60.8) | 134 (18.5) | 1.76  (1.19, 2.61)* | | 0.35  (0.27, 0.45)* |

^*^ These 95% confidence intervals do not include 1

^1^ Adjusted for all other variables in the table

**Table S4.** Prevalence of each autism spectrum disorder severity level with no domain specified by selected characteristics, Autism and Developmental Disabilities Monitoring (ADDM) Network, 2018 and 2020

|  | **Severity Level: No Domain Specified** | | | | | **Adjusted Prevalence Ratio^1^ (95% Confidence Interval)** | |
| --- | --- | --- | --- | --- | --- | --- | --- |
|  | Total | 1 | 2 | 3 | Severity Level 1 | | Severity Level 3 |
|  | n | n (%) | | |  | |  |
| Total | 2,403 | 636 (26.5) | 985 (41.0) | 782 (32.5) |  | | |
| **Sex** |  | | | | | | |
| Male | 1,932 | 518 (26.8) | 804 (41.6) | 610 (31.6) | Reference | | |
| Female | 470 | 117 (24.9) | 181 (38.5) | 172 (36.6) | 1.01  (0.82, 1.23) | | 1.07  (0.90, 1.26) |
| **Race/Ethnicity** |  | | | | | | |
| Non-Hispanic White | 1,461 | 454 (31.1) | 559 (38.3) | 448 (30.7) | Reference | | |
| Non-Hispanic American Indian/Alaska Native | 15 | 3  (20.0) | 4  (26.7) | 8  (53.3) | 0.72  (0.23, 2.25) | | 1.69  (0.83, 3.45) |
| Non-Hispanic Asian | 175 | 29 (16.6) | 83 (47.4) | 63 (36.0) | 0.63  (0.43, 0.92)* | | 1.24  (0.95, 1.63) |
| Non-Hispanic Black | 472 | 89 (18.9) | 211 (44.7) | 172 (36.4) | 0.70  (0.55, 0.89)* | | 1.14  (0.95, 1.38) |
| Hispanic | 198 | 44 (22.2) | 93 (47.0) | 61 (30.8) | 0.73  (0.53, 1.01) | | 1.23  (0.92, 1.63) |
| Non-Hispanic Multiracial | 47 | 7  (14.9) | 23 (48.9) | 17 (36.2) | 0.51  (0.24, 1.09) | | 1.26  (0.77, 2.06) |
| Unknown | 35 | 10 (28.6) | 12 (34.3) | 13 (37.1) | 0.88  (0.45, 1.71) | | 1.11  (0.63, 1.93) |
| **Age** |  | | | | | | |
| 4 years old | 1,083 | 164 (15.1) | 449 (41.5) | 470 (43.4) | Reference | | |
| 8 years old | 1,320 | 472 (35.8) | 536 (40.6) | 312 (23.6) | 1.97  (1.64, 2.38)* | | 0.67  (0.57, 0.78)* |
| **Intellectual Disability** |  | | | | | | |
| No | 984 | 356 (36.2) | 449 (45.6) | 179 (18.2) | Reference | | |
| Yes | 656 | 113 (17.2) | 265 (40.4) | 278 (42.4) | 0.55  (0.44, 0.69)* | | 2.14  (1.76, 2.60)* |
| Unknown | 763 | 167 (21.9) | 271 (35.5) | 325 (42.6) | 0.67  (0.55, 0.82)* | | 1.99  (1.63, 2.44)* |
| **ADDM Surveillance Year** |  | | | | | | |
| 2018 | 926 | 268 (28.9) | 390 (42.1) | 268 (28.9) | Reference | | |
| 2020 | 1,477 | 368 (24.9) | 595 (40.3) | 514 (34.8) | 0.81  (0.69, 0.96)* | | 1.28  (1.10, 1.50)* |
| **Site** |  | | | | | | |
| Arkansas | 154 | 39 (25.3) | 46 (29.9) | 69 (44.8) | Reference | | |
| Arizona | 115 | 28 (24.3) | 52 (45.2) | 35 (30.4) | 0.98  (0.60, 1.60) | | 0.71  (0.47, 1.07) |
| California | 246 | 56 (22.8) | 133 (54.1) | 57 (23.2) | 0.97  (0.64, 1.48) | | 0.57  (0.40, 0.83)* |
| Georgia | 171 | 36 (21.1) | 86 (50.3) | 49 (28.7) | 1.18  (0.74, 1.87) | | 0.52  (0.36, 0.75)* |
| Maryland | 265 | 71 (26.8) | 144 (54.3) | 50 (18.9) | 1.33  (0.89, 1.97) | | 0.37  (0.26, 0.53)* |
| Minnesota | 123 | 46 (37.4) | 47 (38.2) | 30 (24.4) | 1.45  (0.94, 2.23) | | 0.57  (0.37, 0.88)* |
| Missouri | 733 | 154 (21.0) | 250 (34.1) | 329 (44.9) | 1.07  (0.74, 1.34) | | 0.81  (0.61, 1.08) |
| New Jersey | 32 | 8  (25.0) | 15 (46.9) | 9  (28.1) | 1.21  (0.56, 2.59) | | 0.54  (0.27, 1.09) |
| Tennessee | 130 | 56 (43.1) | 47 (36.2) | 27 (20.8) | 1.41  (0.93, 2.13) | | 0.57  (0.36, 0.90)* |
| Utah | 277 | 73 (26.4) | 94 (33.9) | 110 (39.7) | 1.16  (0.78, 1.73) | | 0.79  (0.58, 1.08) |
| Wisconsin | 157 | 69 (43.9) | 71 (45.2) | 17 (10.8) | 1.96  (1.31, 2.93)* | | 0.22  (0.13, 0.38)* |

^*^ These 95% confidence intervals do not include 1

^1^ Adjusted for all other variables in the table
